# Analysis of a Novel Amplitude-Controlled Memristive Hyperchaotic Map and Its Utilization in Image Encryption

**DOI:** 10.3390/s25113388

**Published:** 2025-05-28

**Authors:** Wenfeng Yang, Lingyun Yang, Jian Liu, Rong Li, Yongtao Wang, Ning Chen, Zhaochuan Hu

**Affiliations:** 1 State Key Laboratory of Advanced Design and Manufacture for Vehicle Body, Hunan University, Lushan South Road, Yuelu District, Changsha 410082, China; liujian@hnu.edu.cn (J.L.); jyylirong@hnu.edu.cn (R.L.); wyt409011805@hun.edu.cn (Y.W.); chenning@hnu.edu.cn (N.C.); hzcuni@hnu.edu.cn (Z.H.); 2 Guizhou Aerospace Cloud Network Technology Co., Ltd., No. 1-6, 17th Floor, Unit 2, Building A5, Telford Center, No. 357 Qianlingshan Road, Guiyang National Hi-Tech Industrial Development Zone, Guiyang 550000, China; 3 Department of Automation, Tsinghua University, Haidian District, Beijing 100083, China

**Keywords:** discrete map, hyperchaos, amplitude-controlled, image encryption

## Abstract

In this paper, a global amplitude-controlled discrete hyperchaotic memristive map is designed utilizing the hyperbolic tangent function. This map exhibits fixed points arranged in a line along the *y*-axis, and the stability distributions of these fixed points are delineated based on variations in both the initial conditions of the map and the parameter plane. The dynamic characteristics of the map were examined through the analysis of its 2D dynamics and the largest Lyapunov exponent (LE) distribution. The existence of multistability was robustly confirmed through a comprehensive analysis of the basin of attraction, the spectra of LE that depend on initial values, bifurcation diagrams, and trajectory plots. Additionally, the amplitude of the map can be adjusted both globally and locally through manipulation of the non-bifurcation parameter. Subsequently, a digital circuit powered by a microcontroller was designed to embody the map. In comparison to recent maps, the newly devised map exhibits superior efficacy in the realm of image encryption applications.

## 1. Introduction

In 1971, the concept of the memristor was first proposed by Professor Chua [1]. In 2008, a pioneering research group led by R. Stanley Williams at HP Labs formalized a mathematical framework for memristor systems using Pt/TiO_2_/Pt devices [2]. Memristors, with their data retention and unique nonlinear properties, have emerged as a key focus in academic research and are vital in nonlinear systems [3], the design of chaotic circuits [4,5], neural networks in artificial intelligence [6,7], and brain-inspired computational technologies [8,9]. Memristors are popular for creating chaotic systems with complex dynamics. Unlike traditional nonlinear elements, they offer unique advantages in chaos theory. For example, adding memristors to a Chua’s circuit with an RLC filter or a diode bridge can generate symmetric coexisting chaotic attractors [10]. Yuan introduced a memristor-based chaotic system incorporating a smooth magnetron, which achieves extreme multistability and intricate dynamics, producing multi-scroll hyperchaotic attractors [11]. Additionally, memristors can replicate neuronal firing patterns in memristor-based neurons and neural networks by emulating synaptic behavior and electromagnetic induction [12,13,14].

Creating accurate models of continuous memristors (CMs) is particularly challenging, especially when integrating these components into practical applications [15]. Some research indicates that employing a first-order memristor as a representation of a real device can render the circuit chaotic [16,17].Guseinov studied a second-order memristor system with resistance and capacitance. It has filament length and charge as variables, showing periodic, intermittent, and chaotic behaviors [18]. Valerii Ostrovskii developed a mathematical model for Knowm memristors based on experimental *I-V* curves, incorporating low current effects and cycle variability [19]. Li constructed a memristive Hopfield neural network by introducing two CMs to simulate the autapse of a neuron and the electromagnetic induction effect, which is applied in privacy protection in the Internet of Medical Things [20]. Ding proposed a chaotic memristive neural network integrating two memristors into a traditional Hopfield neural network, which can generate complex hidden multi-wing chaotic attractors [21].

Discrete memristive (DM) chaotic systems have become a research hotspot in recent years due to their unique properties. DM chaotic systems merely need two dimensions [22]. Lately, researchers have presented various DM models based on distinct nonlinear terms, such as trigonometric, absolute value, exponential, hyperbolic tangent, and fractional-order DMs [14,15,23,24]. These models not only enable the creation of chaotic maps but also demonstrate outstanding efficacy. Valerii Yu introduced a new way to create multistability in discrete systems. By applying variable symmetry numerical integration to continuous monostable systems, multiple steady states can be achieved without using complex fractional-order descriptions [25]. Integrating a quadratic charge-governed DM into the Hénon mapping framework notably expands the chaotic domains and significantly enhances the system’s complexity [26]. Furthermore, a 3D hyperchaotic map with two parallel DMs achieves remarkable multistability and hyperchaotic behaviors [27]. Peng introduced a hyperchaotic model incorporating a variable-dimensional interwoven sinusoidal DM, effectively extending the hyperchaotic regions and increasing the system’s intricacy [28]. Yu proposed a 2D memristive map with cosine function, which exhibits global and local amplitude controlling. However, no analysis has been conducted on the complexity of the map [29]. The complexity of the algebraic structure will affect the efficiency of the system in generating chaotic sequences, and low-complexity sequences cannot meet various security encryption requirements. Therefore, there is an urgent need for mapping that has a simple algebraic structure, is highly complex, and can also perform dual amplitude control.

After thorough analysis, we have designed a 2D discrete memristive hyperchaotic map featuring the hyperbolic tangent function (HTMHM). It exhibits multiple stability states and high spectral entropy complexity. Key features are as follows:

1. In terms of dynamic behavior, the system features an infinite number of equilibrium points along the *y*-axis. Their stability is highly sensitive to alterations in model parameters and initial conditions (ICs).
2. The system offers controllable amplitude modulation both locally and globally, and it manifests high spectral entropy (SE) complexity.
3. The STM32 microcontroller verified the feasibility of this mapping, and statistical analyses proved the excellent performance of HTMHM in the field of image encryption.

The remaining sections are arranged as follows. Section 2 describes the formulation of a novel ideal charge-controlled memristor model and its equilibrium points. Section 3 explores the dynamic characteristics of the HTMHM, focusing on equilibrium points, stability analysis, parameter sensitivity, and multistability. In Section 4, the complexity of the HTMHM is assessed through the utilization of spectral entropy (SE). Section 5 details the practical implementation of the HTMHM on digital hardware. Section 6 examines the application of digital image encryption algorithms and includes a statistical analysis of the encryption process. The paper concludes with final observations.

## 2. Discrete Memristive Hyperchaotic Map

### 2.1. Discrete Memristor Model

The charge-regulated DM can be obtained by means of discretizing the continuous memristor. The detailed discretization procedure is presented below [27].

(1) $$\left\{\begin{array}{l} V\left(t_{n}\right)=M\left(q\left(t_{n}\right)\right)i\left(t_{n}\right)\\ \Delta q\left(t_{n}\right)=ki\left(t_{n}\right) \end{array}\right.$$

Equation (1) depicts the state that the CM assumes after the *n*-th sampling event takes place, where Δ*q*(*t_n_*) = *q*(*t_n_*) − *q*(*t_n_*_−1_) and *n* is a positive integer. By determining the expression of *q* for each *n*, a set of equations can be derived, as outlined in Equation (2).

(2) $$\left\{\begin{array}{l} q\left(t_{1}\right)-q\left(t_{0}\right)=ki\left(t_{0}\right)\\ q\left(t_{2}\right)-q\left(t_{1}\right)=ki\left(t_{1}\right)\\ \vdots \\ q\left(t_{n+1}\right)-q\left(t_{n}\right)=ki\left(t_{n}\right) \end{array}\right.$$

The summation of all of the equations, Equation (3), can be yielded.

(3) $$q\left(t_{n+1}\right)=q\left(t_{0}\right)+k\sum_{j=0}^{n}i\left(t_{j}\right)$$

Therefore, the definition of a DM can be described through Equation (4).

(4) $$\left\{\begin{array}{l} v_{n+1}=M(q_{n})i_{n}\\ q_{n+1}=q_{n}+ci_{n} \end{array}\right.$$

Here, *i_n_*, *q_n_*, and *v_n_* denote the discrete samples of the input current *i*(*t*), the internal state variable *q*(*t*), and the outcome *v*(*t*) at the *n*-th repetition, respectively. The term *M*(*q_n_*) signifies the memristance *M*(*q*) at the *n*th iterative cycle

In accordance with the specification of the DM, a new-type discrete memristor featuring a hyperbolic tangent as the nonlinear component was developed.

(5) $$\left\{\begin{array}{l} v_{n}=M(q_{n})i_{n}=\tanh(a\left|q_{n}\right|-h)i_{n}\\ q_{n+1}=c_{1}i_{n}+q_{n} \end{array}\right.$$

**Figure 1:**
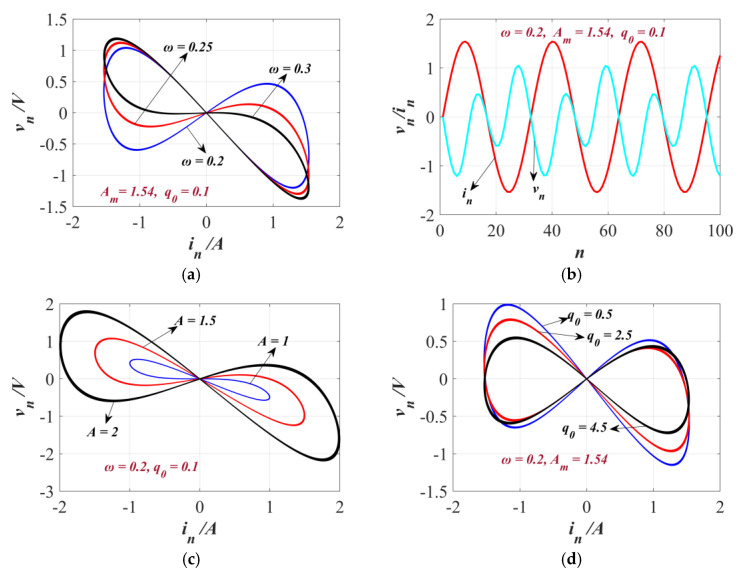
The properties of the DM. (**a**) The PHLs when the input current *i_n_* = 1.54sin(*ω_n_*) and *q*_0_ = 0.1 C at *ω* = 0.15, 0.2 and 0.25 rad/s. (**b**) The series of the memristor under *i_n_* = 1.54sin(0.3*n*) A and *q*_0_ = 0.1 C. (**c**) The PHLs when *i_n_* = *Am*sin(1.54*n*) A and *q*_0_ = 0.1 C at amplitudes *A_m_* = 1 A, 1.5 A, and 2 A. (**d**) The PHLs at *q*_0_ = 0.5 C, 2.5 C, and 4.5 C when *i_n_* = 1.54sin(1.54*n*).

To prove that the developed DM is a generalized memristor, a sinusoidal excitation current source *i_n_* = *Am*sin(*ω_n_*) amperes (where *ω* denotes the angular frequency) was separately applied to this memristor. The key performance of the DM is illustrated in Figure 1.

When the amplitude of *i_n_* is set as *A_m_* = 1.54 A, the initial charge *q*_0_ = 0.1 C, and the angular frequency is specified accordingly, as depicted in Figure 1a, the relationship between *in* and *v_n_* exhibits typical Partial Hysteresis Loops (PHLs), shaped like an italicized “8”. Additionally, the area of the side lobes of this shape diminishes as the *ω* value increases, which is a characteristic of generalized memristors.

With *ω* = 0.2 rad/s, *A_m_* = 1.54 A, and *q*_0_ = 0.1 C held constant, the iteration sequence graph of in and *v_n_* is shown in Figure 1b. This plot clearly highlights the nonlinear nature of the discrete memristor. Likewise, in compliance with the conditions of *q*_0_ = 0.1 C and *ω* = 0.2 rad/s, the amplitude-dependent PHLs are displayed in Figure 1c when *A_m_* is changed to 2 A, 1.5 A, and 1 A, respectively. To delve deeper into the characteristics of the memristor, when *ω* = 0.2 rad/s and *A_m_* = 1.54 A and the initial value of *q*_0_ is set to 1 C, 2 C, and 4 C, respectively, distinct PHLs are generated, as presented in Figure 1d. This confirms the memristor’s multistable nature.

### 2.2. Analysis of Equilibrium Point and Stability

The new memristive map can be obtained according to Equation (5), where *a*, *c*_1_, *k*, and *h* are the parameters of this map.

(6) $$\left\{\begin{array}{l} x_{n+1}=k\tanh(a\left|y_{n}\right|-h)x_{n}\\ y_{n+1}=c_{1}x_{n}+y_{n} \end{array}\right.$$

The proposed memritive map can produce hyperchaos. Generally, the steadiness of chaotic systems is closely related to equilibrium points. Hence, it is assumed that the equilibrium point of HTMHM is *Q* = (*x*∗, *y*∗) [28].

(7) $$\left\{\begin{array}{l} x*=k\tanh(a|y*|-h)\cdot x*\\ y*=x*+y* \end{array}\right.$$

Therefore, the equilibrium point is *Q* = (0, *y*_0_), where *y*_0_ corresponds to the initial value of the state variable *y* in HTMHM. Consequently, *Q* represents an infinite collection of lines along the *y*-axis. The Jacobian matrix *J* evaluated at the equilibrium point *Q* can be expressed by Equation (8).

(8) $$J_{Q=(0,y_{0})}=\left[\begin{array}{cc} k\tanh(a|y_{n}|-h) & 0\\ 1 & 1 \end{array}\right]$$

Hence, the characteristic polynomial can be deduced as

(9) $$P(\lambda )=\lambda^{2}-(k\tanh(a|y_{0}|-h)+1)\lambda +k\tanh(a|y_{0}|-h)$$

Assuming that Equation (9) = 0, the two eigenvalues can be computed as

(10) $$\;\left\{\begin{array}{l} \lambda_{1}=1\\ \lambda_{2}=k\tanh(a|y_{0}|-h) \end{array}\right..$$

In Equation (10), *λ*_1_ = 1, meaning *λ*_1_ stays on the unit circle. As a result, the point *Q* can only be in a state of either critically stable or unstable, and this depends on the value of ∣*λ*_2_∣. When ∣*λ*_2_∣ > 1, *Q* is unstable; conversely, it is in a state of critical stability. When ∣*λ*_2_∣ = 1, the boundary condition for the critical stability of *Q* can be expressed as Equation (11):

(11) $$k\tanh(a|y_{0}|-h)\pm 1=0$$

As stated in Equation (11), the steadiness of point *Q* is closely associated with the parameter *a*, the coupling intensity *k*, and the starting condition *y*_0_. Hence, four scenarios of the stability distribution are analyzed.

**Figure 2:**
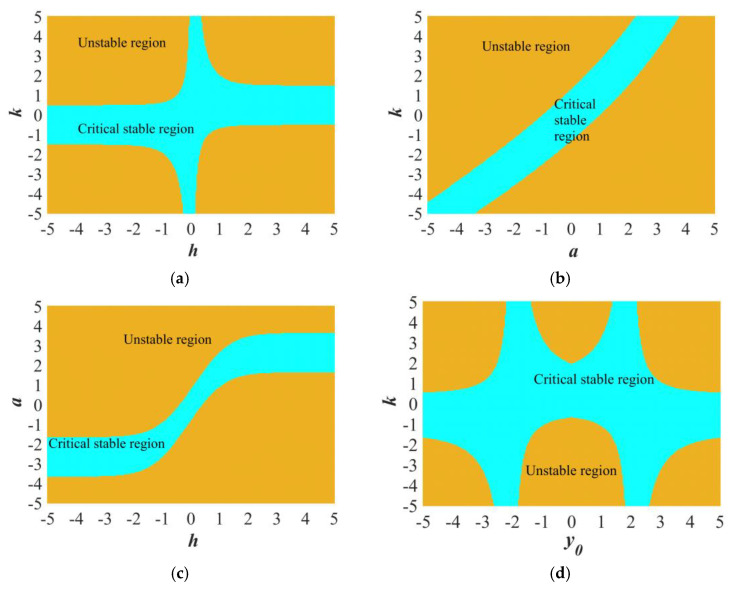
The stability distributions under four distinct combinations of parameters. (**a**) The stability distributions on *k-h*, when IC = (0.1, 0.1), *a* = 0.5. (**b**) The stability distributions on *k-a*, when IC = (0.1, 0.1), *h* = 1. (**c**) The stability distributions on *a-h*, when IC = (0.1, 0.1), *k* = 2.654. (**d**) The stability distributions on *k-a*, when IC = (0.1, 0.1), *h* = 1. (c) The stability distributions on *k-y*_0_, when IC = (0.1, *y*_0_), *k* = 2.654, the parameter *k* = 2.654, *h =* 1.

Case 1: Configuring IC = (0.1, 0.1) and *a* = 0.5, the stability distribution can be depicted on the *k*−*h* plane, where *k* and *h* change ranging within [−5, 5], as illustrated in Figure 2a. The yellow zone denotes the unstable area, suggesting that when the value of the coupling intensity lies within this area, the equilibrium point is unstable. In contrast, the cyan zone signifies the critically stable equilibrium point.

Case 2: Setting IC = (0.1, 0.1) and *h* = 1, the stability spread can be illustrated on the *k*−*a* plane, as presented in Figure 2b. The critically stable area and the unstable area are distinctly differentiated.

Case 3: The initial conditions are set as IC = (0.1, 0.1), the parameter *k* = 2.654, and the stability distribution can be visualized on the *h-a* plane with *h* and *a* varying in the interval [−5, 5]. In Figure 2c, the narrow critical stable region divides the unstable region into two parts.

Case 4: When IC = (0.1, *y*_0_), *a* = 0.5, *h* = 1, and both *k* and *y*_0_ change within the range of [−5,5], the stability pattern can be depicted on the *k*−*y*_0_ plane through the computation of Equation (11), as presented in Figure 2d. The yellow-colored region represents the unstable area, suggesting that when the value of the coupling intensity is in this zone, the equilibrium point is unstable (∣*λ*_2_∣ > 1). In contrast, the cyan zone denotes the critically stable equilibrium point (∣*λ*_2_∣ < 1). The stability pattern is illustrated on the *k*−*y*_0_ plane.

## 3. Dynamic Behaviors

### 3.1. Dynamical Behavior Relay on Parameters

Based on the previous discussion regarding stability distribution, the dynamic distribution of the newly created HTMHM can be described by the dynamic characteristics and the distribution of the largest LEs on the 2D parameter plane via the choice of suitable parameters and ICs. This enables further differentiation and clarification of the map’s dynamic characteristics. The dynamic characteristics of the HTMHM were examined using three sets of the three adjustable parameters, specifically, *a*, *h*, and *k*.

**Figure 3:**
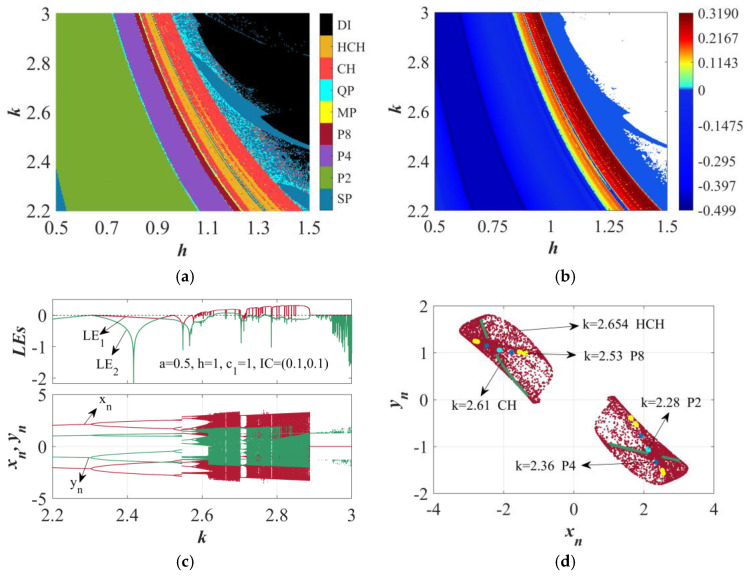
The dynamics of HTMHM when *a* = 0.5 and IC = (0.1, 0.1). (**a**) The 2D distribution of dynamic behaviors in the *k-h* plane. (**b**) The 2D distribution of the largest LE in the plane of *k-h*. (**c**) The LE spectra and bifurcation diagram when *k* = 2.654. (**d**) The attractors when *k* takes typical values.

When the IC is (0.1, 0.1), *a* = 0.5, and *k* and *h* change within the interval of [0.5, 1.5], the pattern of dynamic behaviors is illustrated in Figure 3a. Different dynamic behaviors are marked by regions of various colors and differentiated according to LE values and the quantity of oscillation cycles. The hyperchaotic attractor is associated with the orange region, where the map displays two positive LE values, denoted as HCH. The red-colored region is the chaotic area with one positive LE, which is marked as CH. Furthermore, the region colored steel blue contains a stable point attractor, denoted as SP, while the black-filled divergent behavior is indicated as DI. Meanwhile, several distinct colors are utilized for the areas of period-2, period-4, period-8, multi-period, and quasi-periodic patterns. They are marked as P2, P4, P8, MP, and QP. Figure 3b presents the 2D distribution of the largest LE on the *k*−*h* plane. The blue zone indicates periodic dynamics, given that the largest LE does not exceed zero. Cyan, green, yellow, and red shadings are used to mark the chaotic and hyperchaotic regions, each of which indicates that the largest LE has positive values. The white color is used to signify the divergent behavior of the HTMHM. Through comparison of Figure 3a,b, the 2D distribution of the largest LE efficiently elucidates the configuration of dynamical behaviors. It is evident from Figure 3a that altering the parameters results in strikingly different dynamic behaviors. In order to attain a more profound grasp of how the dynamic behavior of HTMHM evolves, a bifurcation diagram and the LE spectra that are affected based on a lone parameter are drawn.

Using *h* = 1 held constant, the bifurcation plot and the spectra related to LEs for the parameter *k* within the interval [2.2, 3] are presented in Figure 3c. The HTMHM transitions into chaotic and hyperchaotic states via period-doubling bifurcation. As *k* varies, when *k* = 2.3, the HTMHM bifurcates from the P2 to the P4 state and then further bifurcates to the P8 state at *k* = 2.524. Subsequently, bifurcation windows for P8, MP, and QP states emerge within the range of *k* ∈ [2.58, 2.8]. The uppermost part of Figure 3c shows that for *k* ∈ [2.2, 2.58] ∪ [2.7, 2.71] ∪ [2.784, 2.786], the values of the LE are not more than zero, which signals that the HTMHM remains in a periodic state throughout this particular scope. While *k* ∈ (2.58, 2.63] ∪ (2.71, 2.785] ∪ (2.787, 2.88], there is only a single LE value that is greater than zero, which implies that chaotic behavior emerges within this specific interval. Two positive LE values are detected within the range of *k* ∈ [2.629, 2.66] ∪ [2.665, 2.7] ∪ [2.764, 2.767], which is a sign of hyperchaotic behavior. When *k* is greater than 2.88, the state of the mapping promptly returns to a settled position. Figure 3d presents the attractors within the *x−y* plane, which are associated with the typical values of *k*.

### 3.2. Control of the Amplitude

**Figure 4:**
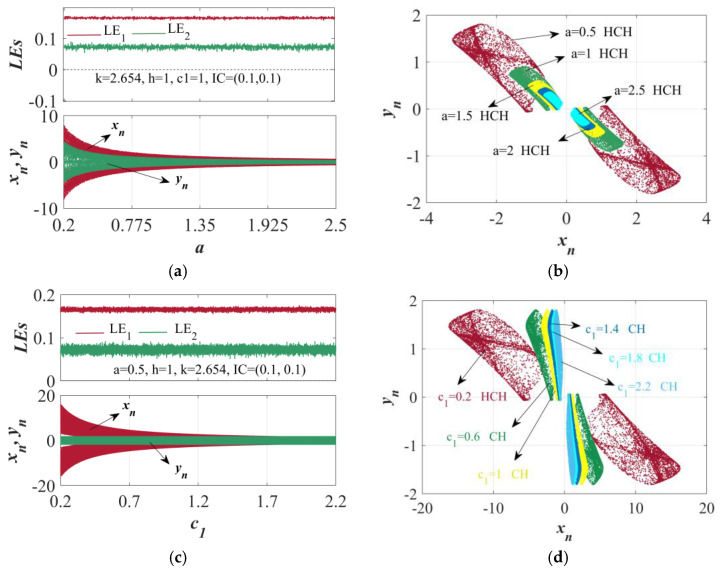
When the parameters are configured as *k* = 2.654, *h* = 1, and *c*_1_ = 1, and the starting condition is IC = (0.1,0.1), the dynamic behavior of these parameters hinges on parameter *a*. (**a**) The LE spectrum and the bifurcation diagram. (**b**) The attractor in the *x*−*y* plane. When the parameters are *k* = 2.654, *h* = 1, and *a* = 0.5, and the starting condition is IC = (0.1,0.1), (**c**) the LE spectrum and the bifurcation diagram. (**d**) Corresponding to a point of stability within the *x*−*y* plane.

Apart from the coupling intensity of the memristor being able to control the dynamic behavior in HTMHM, parameters *a* and *c*_1_ can likewise adjust the dynamic features of the map. When *k* = 2.654, *h* = 1, *c*_1_ = 1, and IC = (0.1, 0.1), the LE spectra and bifurcation diagrams within the range of *a* ∈ [0.2, 2.5] were computed, as shown in Figure 4a. Irrespective of how parameter *a* varies, the LE values do not experience substantial changes, and there are consistently two positive LEs. Yet, based on the bifurcation diagram at the bottom part of Figure 4a, it is evident that no bifurcation occurs as *a* changes. Owing to the increment in *a*, the amplitudes of the map in the *x* and *y* directions diminish to some degree. Thus, *a* is called a non-bifurcation parameter, and it is also termed a global amplitude control parameter. As illustrated in Figure 4b, the attractors corresponding to different values of a can be drawn in the *x*−*y* plane. It is evident that modifying *a* does not cause a transformation in the state of the map. Instead, it simply modifies the magnitude of the attractor.

In this map, apart from *a* being a non-bifurcation parameter, *c*_1_ is also a non-bifurcation parameter. To analyze the impact of parameter *c*_1_ on the mapping, with *k* = 2.654, *h* = 1, *a* = 0.5, and IC = (0.1, 0.1), the bifurcation diagrams and LE spectra within the range of *c*_1_ ∈ [0.2, 2.2] were calculated, as shown in Figure 4c. As *c*_1_ varies, there is no alteration in the LE, and the two LE values consistently remain greater than 0 and relatively constant. A peculiar phenomenon emerges in the bifurcation diagram at the bottom of Figure 4c, where the amplitude in the *x* direction decreases as *c*_1_ increases, while the amplitude in the *y* direction remains unchanged. Hence, parameter *c*_1_ can be termed a local amplitude control parameter. Figure 4d presents the attractors corresponding to typical values of *c*_1_. It can be observed that the change of parameter *c*_1_ does not induce a change in the mapping state but only regulates the scale of the attractor in the *x* direction.

### 3.3. Multistability

Multistability refers to the coexistence of multiple attractors in phase space under varying ICs [30]. This phenomenon is identifiable through attraction basins displaying multi-colored regions, each corresponding to distinct stable states governed by system dynamics and ICs. Multistability is confirmed when three or more color-coded attractor regions coexist [31]. Additionally, stability analysis via LE variations and period numbers can reveal multistable behavior. Here, we investigate multistability in the HTMHM using a 2D basin of attraction (*x*_0_, *y*_0_ ∈ [−4.8, 2.4]) under fixed parameters (*a* = 0.5, *k* = 2.654, *c*_1_ = 1, *h* = 1).

**Figure 5:**
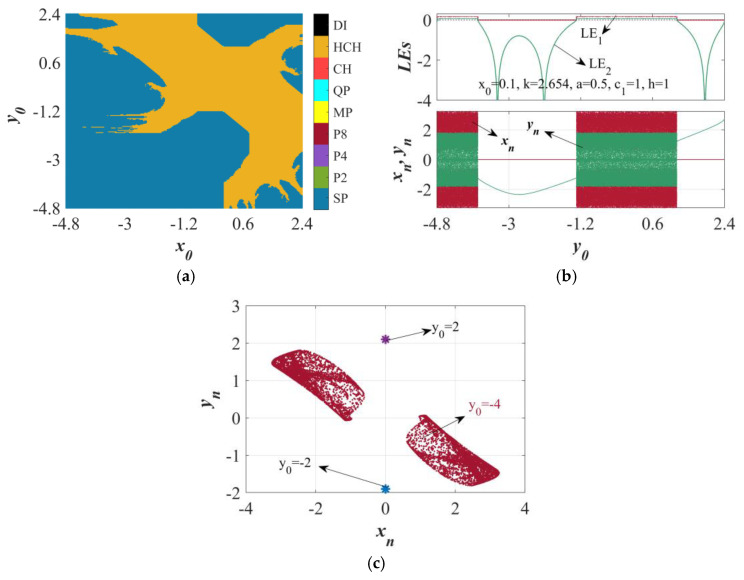
When the parameters a = 0.5, *k* = 2.654, *h* = 1, and *c*_1_ = 1 and the initial condition IC = (*x*_0_, *y*_0_) are specified, novel mapping exhibits extreme multistability. (**a**) The two-dimensional local attractor region in the *x*_0_−*y*_0_ plane. (**b**) The LE spectrum and the bifurcation diagram when *x*_0_ = 0.1. (**c**) The co-existing attractors in the *x_n_*−*y_n_* plane.

As shown in Figure 5a, color-separated basins indicate coexisting stable points and hyperchaotic attractors. Dynamic evolution analysis (*x*_0_ = 0.1, *y*_0_ ∈ [−4.8, 2.4]) in Figure 5b reveals three phases: hyperchaos at *y*_0_ < −3.773, intermittent chaos with a stable point (−3.773 < *y*_0_ < −1.308), and stable point dominance after *y*_0_ > 1.208 via tangent bifurcations. Figure 5c further validates the heterogeneous coexistence of hyperchaotic and stable point attractors under these parameters.

## 4. Energy and Complexity

Energy analysis facilitates comprehensive comprehension of the system’s dynamic characteristics, stability attributes, complexity dimensions, and susceptibility to perturbations in initial conditions and parametric alterations. The energy function associated with a 2D chaotic map may be formally defined as

(12) $$H_{n}=x_{n}^{2}+y_{n}^{2}$$

To calculate the total energy of the system over a series of time steps, we can sum the energy at each time step.

(13) $$H_{\mathrm{total}}=\sum_{n=0}^{N-1}H_{n}=\sum_{n=0}^{N-1}\left(x_{n}^{2}+y_{n}^{2}\right)$$

The average energy can be obtained by dividing the total energy by the number of time steps *N*.

(14) $$<H_{n}>=\frac{1}{N}\sum_{n=0}^{N-1}\left(x_{n}^{2}+y_{n}^{2}\right)$$

**Figure 6:**
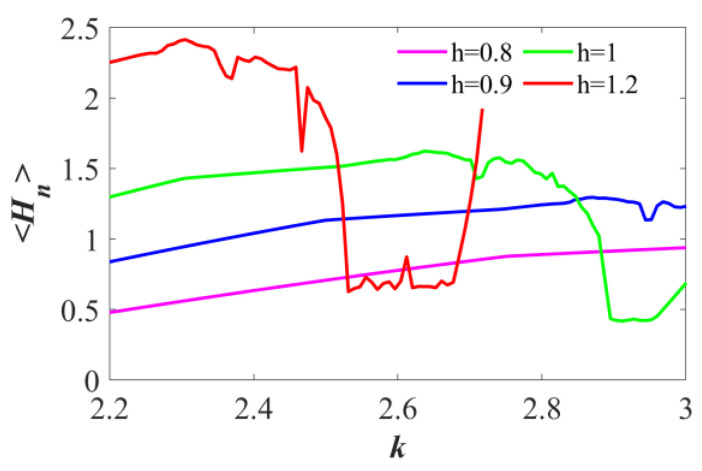
The average energy of HTMHM under the conditions of *a* = 1, *c*_1_ = 1, *h* = 1, and IC = (0.1, 0.1).

Figure 6 illustrates the average energy spectrum as the parameter *k* varies in the interval of [2.2, 3] for different values of *h*. When fixing *h* = 1, *a* = 1, *c*_1_ = 1, and IC = (0.1, 0.1), the average energy <*H_n_>* = 1.6292.

Complexity is a measure used to assess the similarity between a wild and irregular sequence and a whimsical sequence [31]. Higher complexity indicates greater randomness in the chaotic sequence. The chaotic properties of the system are examined by analyzing the interactions among system parameters. Using the “turbo” mode in MATLAB R2021b, the relationships between parameter variations are depicted through multi-colored contour graphs. The complexity of the new mapping is determined using the SE complexity measurement algorithm.

**Figure 7:**
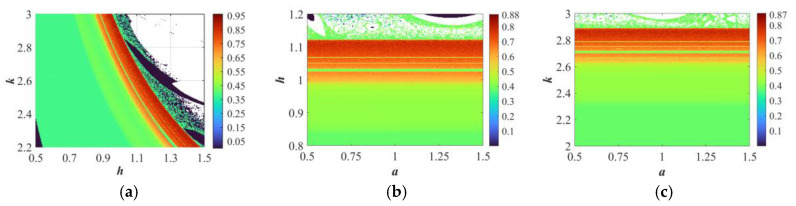
The HTMHM relates to the SE complexity distribution graph for the *k-h* under the conditions of *a* = 1, *c*_1_ = 1, and IC = (0.1, 0.1). (**a**) *k* vs. *h*. (**b**) *a* vs. *h*. (**c**) *a* vs. *k*.

When the parameters are set to *a* = 0.5, *c*_1_ = 1, and the initial condition IC = (1.0, 1.0), with *k* and *h* varied within the range of [0.5, 3], the SE complexity contour graph for HTMHM is displayed in Figure 7a. Here are the areas that exhibit varying degrees of complexity, with some characterized by high complexity and others by low complexity distinctly separated through varied colors. The SE complexity involvedness of 0.952 at (*k*, *h*) = (2.654, 1). Figure 7b,c show the SE complexity distribution of *a* vs. *k* and *a* vs. *h*, respectively. According to the 2D complexity distribution chart with the dynamic and largest LE distribution, high-complexity regions align with chaotic regions and areas where LE is more than 0. Conversely, the regions of diminished complexity are associated with zones exhibiting periodic or quasi-periodic behavior, as well as areas where the LE is less than or equal to zero, as depicted in the dynamic distribution and LE distribution diagrams.

## 5. Implementation with Digital Hardware

Two approaches exist for implementing anarchic systems and mappings: pure analog circuitries and digital circuits centered around microcontrollers. It is the parasitic capacitance in analog circuits that complicates the process of setting ICs. In contrast, microcontroller-based digital circuits allow for flexible configuration of parameters and ICs through programming. Additionally, within the microcontroller chip, core computations are implemented, simplifying circuit design and addressing the limitations of analog circuits [32]. The key principle behind digital hardware construction for discrete chaotic phenomena maps is the use of the current cycle’s output as the next cycle’s input and executing sufficient iterations. Dissimilar to continuous chaotic systems [33], discrete chaotic maps are free from the necessity of undergoing a discretization process, eliminating uneasiness about the deterioration in chaotic characteristics.

**Figure 8:**
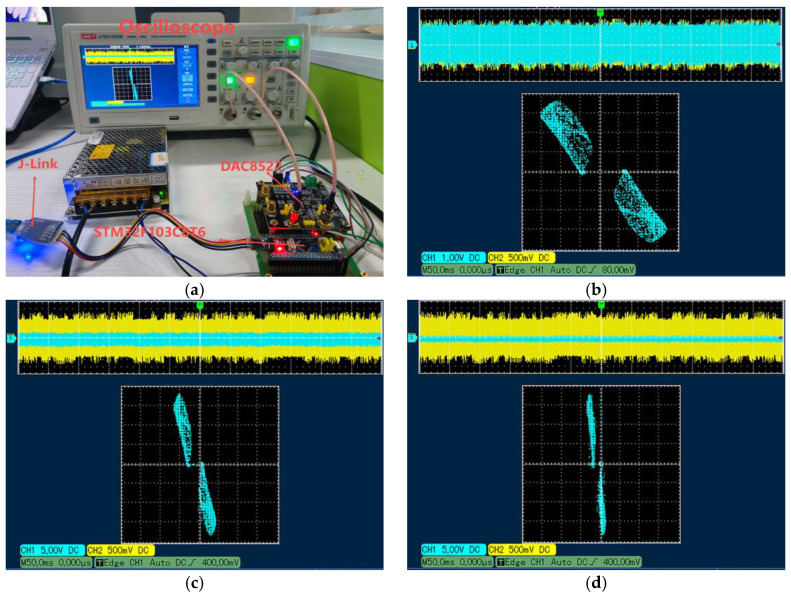
Digital circuit implementation. (**a**) The actual experimental platform. (**b**) *a* = 0.2. (**c**) *a* = 0.6. (**d**) *a* = 1.4.

The digital implementation platform includes a personal computer equipped with the development tool “Keil μVision 5”, a ±15 V precision-regulated power supply, a microcontroller STM32F103C8T6 with the Cortex-M3 core (72 MHz clock frequency, 64 KB Flash), which has a typical operating power consumption of 36 mA at 72 MHz, supports DMA to enhance peripheral throughput efficiency, and is suitable for medium and low load embedded control scenarios, an SWD adapter, a fast bipolar D/A conversion module DAC8552, and a dual-trace oscilloscope, as well as some other peripheral circuits. The experimental setup is shown in Figure 8a. The mathematical model of HTMHM is programmed in C language, and the compilation process occurs inside of Keil *μ*version 5 to produce an executable file for the microcontroller containing necessary elements, such as the ‘.axf’ or ‘.hex’ extension. Following the previous steps, it is downloaded onto the microcontroller via the J-link. When the download of the program comes to an end, the microcontroller brings forth digital signals that match the state variables *x* and *y*. These digital signals are transmitted to the DAC8552 using the SPI communication interface. Within the DAC8552, the digital representations of *x* and *y* are transformed into dual-polarity analog signals, which are then dispatched to channels A and B of the oscilloscope for distinct analytical assessments. Using the parameters and ICs from Section 3.2, the oscilloscope-captured phase portraits projected onto the *x-y* plane are illustrated in Figure 8b–d. Analysis of the oscilloscope waveforms reveals strong alignment with the numerical simulation results in Figure 8d. This close correspondence confirms the feasibility of the presented HTMHM, as well as its aptitude for practical realizations.

## 6. Application in Image Encryption

### 6.1. NIST Test

The NIST SP800-22 test suite, developed by the National Institute of Standards and Technology (NIST), consists of 15 rigorous statistical tests designed to evaluate the randomness of pseudorandom number sequences. Each test examines different aspects of non-random patterns in the sequence and produces one (or, in rare cases, multiple) *p*-value(s). The significance level (α) is set at 0.01, defining the threshold for rejecting the randomness hypothesis. A sequence passes an individual test if its *p*-value exceeds α. However, following the criteria outlined in reference [32], *p*-values that meet or exceed α require further validation. Specifically, 256 *p*-values from each test are subjected to a chi-square goodness-of-fit test, generating a secondary *p*-value (*p*-value*_T_*). Both *p*-value*_T_* and the overall pass rate serve as critical indicators for assessing sequence randomness. A sequence is considered uniformly distributed if *p*-value*_T_* ≥ 0.0001. Based on the above framework and the computational formula provided in reference [32], a pass rate of ≥ 0.98906 constitutes statistically significant evidence of randomness. The section below Table 1 presents the final test results of two pseudorandom sequences generated by the HTMHM system. All evaluation metrics meet the required pass criteria, confirming that both sequences successfully pass the rigorous NIST SP800-22 statistical test suite. These results demonstrate that the pseudorandom sequences derived from HTMHM’s hyperchaotic system exhibit strong randomness and uniformity, highlighting their potential advantages for applications in image encryption and secure communication systems.

### 6.2. Image Encryption Algorithm

**Figure 9:**
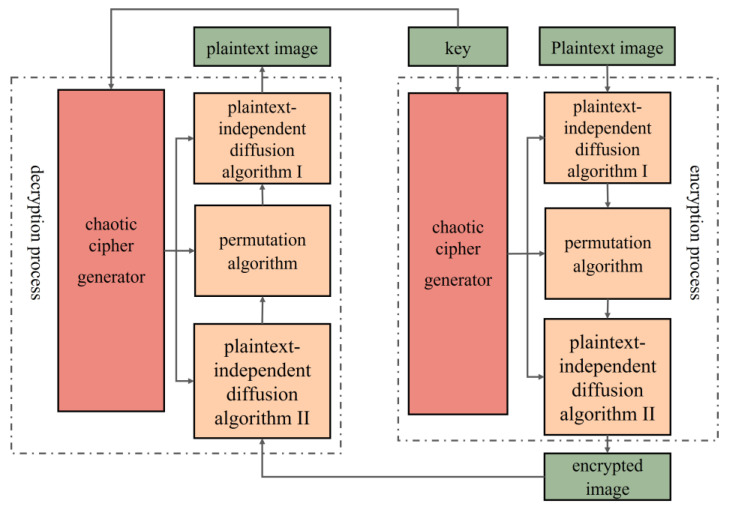
Flowchart of the image encryption algorithm.

In this paper, we employ an image encryption algorithm that incorporates plaintext-related scrambling, as illustrated in Figure 9. The specific steps are detailed below.

Step 1: Flatten the 2D unencoded visual data *P* into a one-dimensional vector of size *M×N*, where *M* is the row count and *N* is the column count of the matrices for the clear and unencrypted images.

Step 2: Two integers, (*r*_1_, *r*_2_), are randomly selected from the interval [0, 255], combined with the initial values *x*_0_ and *y*_0_ of the chaotic system to form the encryption and decryption key of the system, denoted as *K* = (*x*_0_, *y*_0_, *r*_1_, *r*_2_).

Step 3: Two pseudorandom sequences generated by the chaotic system are converted into six independent integer sequences, with each element value ranging from 0 to 255. These integer sequences are then sequentially assigned to the two *M × N* matrices, *X* and *Y*.

Step 4: Using the diffusion algorithm I and the random matrix *X*, the pixel values at corresponding positions in the initial plaintext image *P* are transformed, thereby converting it into a new matrix, *P*_1_. Its processing process is as shown in Equation (15).

(15) $$\left\{\begin{array}{c} P_{1}(1,1)=\mathrm{mod}\{P(1,1)+X(1,1),256\}\\ \begin{array}{c} P_{1}(i,1)=\mathrm{mod}\{P(i,1)+\sum_{k-1}^{N}X(i-1,1),256\};(M\geq i\geq 1)\\ P_{1}(i,j)=\mathrm{mod}\{P(i,j)+P(i,j-1)+X(i,j),256\};(M\geq i\geq 1,N\geq j\geq 1) \end{array} \end{array}\right.$$

where *i* ∈ [1, *M*] and *j* ∈ [1, *M*] and *P*(*i*, *j*) indicates the grayscale value of the image *P* at the given coordinate (*i*, *j*).

Step 5: So as to break the correlation among adjacent pixel points in the original imagery, two integers, *k* and *l,* are generated through calculations using random matrices *Z*, *W*, *U*, and *V* through Equation (16). Subsequently, the image matrix *P*_1_ is traversed in a specific sequence, and the pixel’s value at the given spot *P*_1_(*i*, *j*) is interchanged with that at position *P*_1_(*k*, *l*), obtaining the scrambled image *P*_2_.

(16) $$\left\{\begin{array}{c} H=\left\{\sum_{k-1}^{N}W(a,k)\right\},a\in [1,M]\\ L=\left\{\sum_{k-1}^{N}Z(a,k)\right\},b\in [1,M]\\ k=\mathrm{mod}\left\{U(i,j)+H[C(i,j)],M\right\}+1\\ l=\mathrm{mod}\{V(i,j)+H[C(i,j)],M\}+1 \end{array}\right.$$

A reversible Arnold matrix TT is employed for pixel shuffling to guarantee the encryption’s reversibility. The fixed pixel *P*_2_(*i*,*j*) is exchanged with the random pixel *P*_2_(*r*,*s*) to generate the ciphertext image *P*_3_, where *r* and *s* can be determined through Equation (17)

(17) $$\left[\begin{array}{c} r\\ s \end{array}\right]=\mathrm{mod}\left\{T\cdot \left[\begin{array}{c} i\\ j \end{array}\right],\left[\begin{array}{c} M\\ N \end{array}\right]\right\}+\left[\begin{array}{c} 1\\ 1 \end{array}\right]$$

The inverse matrix of *T*, represented as *T*^−1^, assumes a crucial part in the decryption operation. The formulations of *T* and *T*^−1^ are presented in Equation (18).

(18) $$\begin{array}{l} \left\{\begin{array}{l} T=\left[\begin{array}{cc} 1 & Z(i,j)\\ W(i,j) & Z(i,j)\cdot W(i,j)+1 \end{array}\right]\\ T^{-1}=\left[\begin{array}{cc} Z(i,j)\cdot W(i,j)+1 & -Z(i,j)\\ -W(i,j) & 1 \end{array}\right] \end{array}\right. \end{array}$$

Step 6: Execute a subsequent diffusion action upon the image *P*_3_ with the random matrix. As opposed to step 4, this process is conducted in reverse, starting from the last pixel of the image and propagating forward, eventually obtaining the encrypted image *P*_4_.

(19) $$\left\{\begin{array}{c} P_{4}(M,N)=\mathrm{mod}\{P_{3}(M,N)+Y(M,N),256\}\\ P_{4}=(i,N)=\mathrm{mod}\left\{P_{3}(i,N)+\sum_{k-1}^{N}P_{4}(i+1,k)+Y(i,N),256\right\};(M>i\geq 1)\\ P_{4}(i,j)=\mathrm{mod}\{P_{3}(i,N)+P_{4}(i,j+1)+Y(i,j),256\};(M>i\geq 1,N>j\geq 1) \end{array}\right.$$

The steps involved in decrypting an image are precisely the opposite of those in the image encryption process.

**Figure 10:**
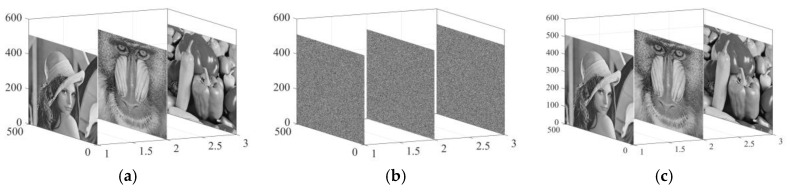
Encryption and decryption outcomes: (**a**) original images. (**b**) Encryption results. (**c**) Decryption results.

Encryption and decryption tests were performed using three well-known images (Lena, Baboon, and Peppers). The data come from the USC-SIPI image database, which is accessible to the public. The outcomes of this algorithm are presented in Figure 10a, which presents the starting images, whereas the encrypted counterparts corresponding to them are showcased in Figure 10b. After that, the display of the decrypted images takes place in Figure 10c. It is noticeable from Figure 10b that the encrypted image gives the impression of being both randomly arranged and thoroughly scrambled, effectively concealing the information of the original image.

### 6.3. Statistical Analysis of Encryption

Several traditional numerical test methods are effective tools for assessing the functionality of encryption systems and the merit of their results. Consequently, histograms, the *χ*^2^ test, entropy related to information, the mean squared error (MSE), the top value of the signal-to-noise ratio (PSNR), the dependency coefficient, the pixel change rate (NPCR), the uniform average change intensity (UACI), and the block average change intensity (BACI) have been employed to evaluate the advanced encryption system under discussion and its results. Furthermore, with the implementation of salt and pepper noise and cropping manipulations, the resilience of the recommended encryption algorithm to noise-related issues and data loss has been explored.

Plaintext sensitivity analysis refers to the use of the same key to encrypt two plaintext images that differ slightly using an image encryption system, resulting in two corresponding ciphertext images. The differences between these two ciphertext images are compared. If the differences are significant, the image encryption system is said to have good plaintext sensitivity; if the differences are small, the system is said to have weak plaintext sensitivity, which generally cannot resist chosen plaintext attacks or known plaintext attacks. The two so-called plaintext images that differ slightly can be obtained through slight modification of the value of one or several pixel points in a given plaintext image. For example, randomly selecting a pixel point (*i*, *j*) from a plaintext image *P*_1_, changing its value to *P*_1_(*i*, *j*) + 1, and taking the value modulo 256 will yield a plaintext image *P*_2_ that has a minor difference from *P*_1_. The NPCR, UACI, and BACI metrics are calculated 200 times, and the mean values of these metrics across all iterations are subsequently determined. The test outcomes presented in Table 2 demonstrate that the proposed image encryption system exhibits strong plaintext sensitivity.

**Figure 11:**
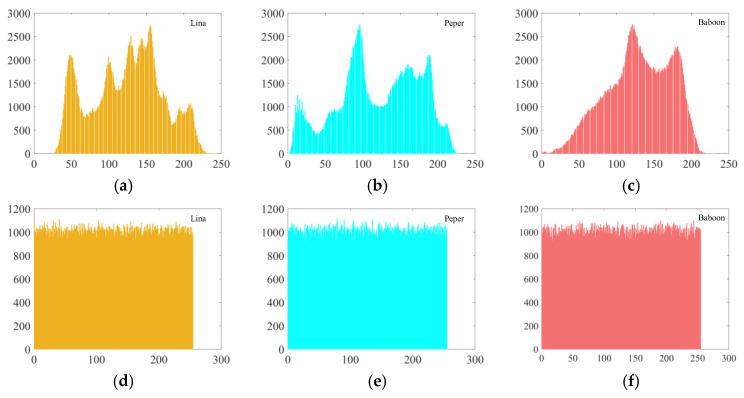
Histograms: (**a**–**c**) are the histograms of the plaintext images, and (**d**–**f**) are the histograms of the encrypted images.

Histograms allow for the assessment of the distribution pattern of pixel values, which are used to describe how the image information is distributed. By making a comparison of the histograms of the initial images in Figure 11a,b with those of the encrypted images in Figure 11c,d, the histogram transitions from a fluctuating to a uniform pattern after encryption.

The *χ*^2^ test is a commonly employed technique for evaluating histogram differences between original and encrypted images. With a significance level α = 0.05, the statistic for an image in grayscale mode with 256 possible gray levels is *χ*^2^0.05(256) = 293.24783. The results are presented in Table 3. Notably, the *χ*^2^ values for the three original images are considerably higher than 293.24783, while those for their encrypted counterparts are all below 293.24783, suggesting that the distributions in Figure 11d–f are nearly uniform [33].

Informational entropy (IE) content measures the doubtfulness regarding the data within images; higher magnitudes suggest an enhanced state of random variation and lack of order within the picture. As a result, it stands as a significant indicator when it comes to assessing how secure the encrypted results are. The technique for determining information entropy is given in Equation (20).

(20) $$H(x)=-\sum_{i=1}^{L}P(x_{i})\log_{2}P(x_{i})$$

Here, *L* represents the gray level associated with the image. Inside of the encryption mechanism we have implemented, *L* = 256; *P*(*xi*) stands for the probability of the pixel’s value being *x_i_*. Regarding an image with a total of 256 grayscale gradations, the theoretical worth of information entropy *H* is eight. Thus, when the entropy of the encrypted image comes closer to the figure of eight, the more secure the encryption scheme. Presented in Table 4 are the results from the IE test for the encryption system we proposed. Plainly, the entropies related to the three encrypted images are very near the theoretical magnitude. Furthermore, comparisons with references [22,34,35,36] establish the elevated level of randomness and disorder present in the encrypted images.

MSE quantifies the average squared difference in pixel values between two images. Consequently, a higher MSE with more dissimilarity existing between the unencrypted image and the corresponding encrypted one implies higher-quality encryption results. Typically, an MSE value greater than 30 dB [37] indicates a significant disparity between the two images. The formula for computing MSE is as follow:

(21) $$MSE=\frac{1}{M\cdot N}\sum_{i=1}^{M}\sum_{j=1}^{N}{\left[P(i,j)-Q(i,j)\right]}^{2}$$

In this context, *P*(*i*,*j*) and *Q*(*i*,*j*) refer to the values related to the pixels of the original and encrypted images at coordinates (*i*,*j*), individually. The dimensions of the image grid are given by *M* rows and *N* columns. To assess encryption quality, the peak signal-to-noise ratio (PSNR) serves as a critical metric, which can be formulated as [38]

(22) $$PSNR=20\log_{10}(\frac{P_{\max}}{\sqrt{MSE}})$$

Here, *P*_max_ denotes the maximum magnitude of the pixel value in the image. In the course of our testing, *P*_max_ is 255. Unlike MSE, a lower PSNR proves better encryption capabilities; this is clear when considering the inverse correlation between the PSNR and the MSE. The MSE and PSNR outcomes for both the original and encrypted renditions of the images Lena, Baboon, and Peppers are displayed in Table 5. It turns out that the MSE values for all three images exceed 30 dB, and the PSNR value with respect to the recommended encryption plan are comparable to or even lower than those reported in references [22,34,35,36]. These numerical results further validate the robustness of the encryption outcomes.

The correlation coefficient serves as a crucial metric when it comes to evaluating the interrelation of neighboring pixels within an image. The correlation coefficient, denoted as *C_xy_*, that exists between adjacent pixels is formulated as [39,40,41]

(23) $$C_{xy}=\frac{\sum_{i=1}^{n}\left(x(i)-X)\cdot ((y(i)-Y)\right)}{\sqrt{\sum_{i=1}^{n}{\left(x(i)-X\right)}^{2}}\cdot \sqrt{\sum_{i=1}^{n}{\left(x(i)-Y\right)}^{2}}}$$

where in $X=\frac{1}{N}\sum_{i=1}^{N}x(i)$ and $Y=\frac{1}{N}\sum_{i=1}^{N}y(i)$; in this scenario, *X* and *Y* show the arithmetic mean values of the pixel sequences *x*(*i*) and *y*(*i*), respectively. Table 6 provides the correlation coefficients for plaintext images alongside their encrypted counterparts in four different directions. The plaintext images show high correlation coefficients, often nearing one across all directions. In contrast, the encrypted images produced through our encryption technique demonstrate substantially lower correlation coefficients, which are nearly zero in every direction. This suggests that there is minimal relationship of neighboring pixels amidst the encrypted image dataset. Furthermore, a comparison of our findings with those reported in references [22,34,35,36] supports the robustness and effectiveness of our encryption method.

Key sensitivity and spatial analysis: NPCR (Number of Pixel Changing Rate), UACI (Unified Average Changing Intensity), and BACI (Bit Average Change Intensity) are crucial parameters for judging the effectiveness pertaining to an encryption technique system. These metrics serve to conduct a quantitative analysis of the disparities between a pair of images. In the case of two completely different images, the optimal figures for the Number of Pixel Change Rate (NPCR), Unified Average Changing Intensity (UACI), and Bit-Average Changing Intensity (BACI) are 99.6094%, 33.4635%, and 26.7712%, in that order. These metrics are assessed using pairs of images with identical dimensions, where images encrypted as test samples are composed of those with dissimilar keys. When conducting the evaluation of NPCR, UACI, and BACI, this approach facilitates the exploration of the key sensitivity aspect of the encryption system.

During our experimental endeavors, the initial values *x*_0_ and *y*_0_ for the chaotic mapping, being integral parts of the key, were defined as variables that can be controlled. Regarding the system parameters (*a*, *k*, *c*_1_, *h*), they were designated as (0.5, 2.654, 1, 1) (0.5, 2.654, 1, 1). During testing, the initial values *X*_0_ and *Y*_0_ were randomly chosen from the key interval. The unit of variation for the private key was configured as 10^−13^. Subsequently, several sets of keys were produced by utilizing (*x*_0_, *y*_0_) = (*X*_0_*+n*⋅10^−13^, *Y*_0_+*m*⋅10^−13^)(*x*_0_, *y*_0_) = (*X*_0_+*n*⋅10^−13^, *Y*_0_+*m*⋅10^−13^), where *n* and *m* are integers, to create multiple encrypted images, resulting in 600 key sets where *x*_0_ and *y*_0_ change continuously. To evaluate the key sensitivity, the NPCR, UACI, and BACI metrics were computed based on the ciphertext images before and after consecutive key alterations. As depicted in Table 7, the final outcome was determined by taking the average of all of the test findings.

It is evident that the experimental results closely align with the theoretical values. Notably, some of the results even surpassed the expected benchmarks. From this, it can be inferred that subtle modifications to the key have the potential to induce substantial transformations in the encrypted image. This, in turn, validates the encryption system’s responsiveness to key changes.

It is clear from these tests that the images of the ciphertext before and after key adjustment (as small as 10^−13^) are completely different, highlighting the extraordinary sensitivity of the system to key changes. Therefore, 10^−13^ can be taken as the most minuscule measurable entity in determining the key space of the encryption system. For the purpose of ensuring that the chaotic system keeps its chaotic nature within the key space, the scopes of *x*_0_, *y*_0_, and *c*_1_ are set at [0, 5], [0, 1.5], and [4.3, 5], respectively. Within these ranges, with *x*_0_ in combination with other variables, it can bring about highly random chaotic sequences, and the combination of *y*_0_ and *z*_0_ can also generate such sequences. The overall key space is calculated as (10^9^ × 10^13^ × 1.5 × 10^13^ × 0.7 × 10^13^) = 1.05 × 10^40^. This value exceeds 2100, implying that the put-forward encryption system can successfully repel brute-force onslaughts [42].

**Figure 12:**
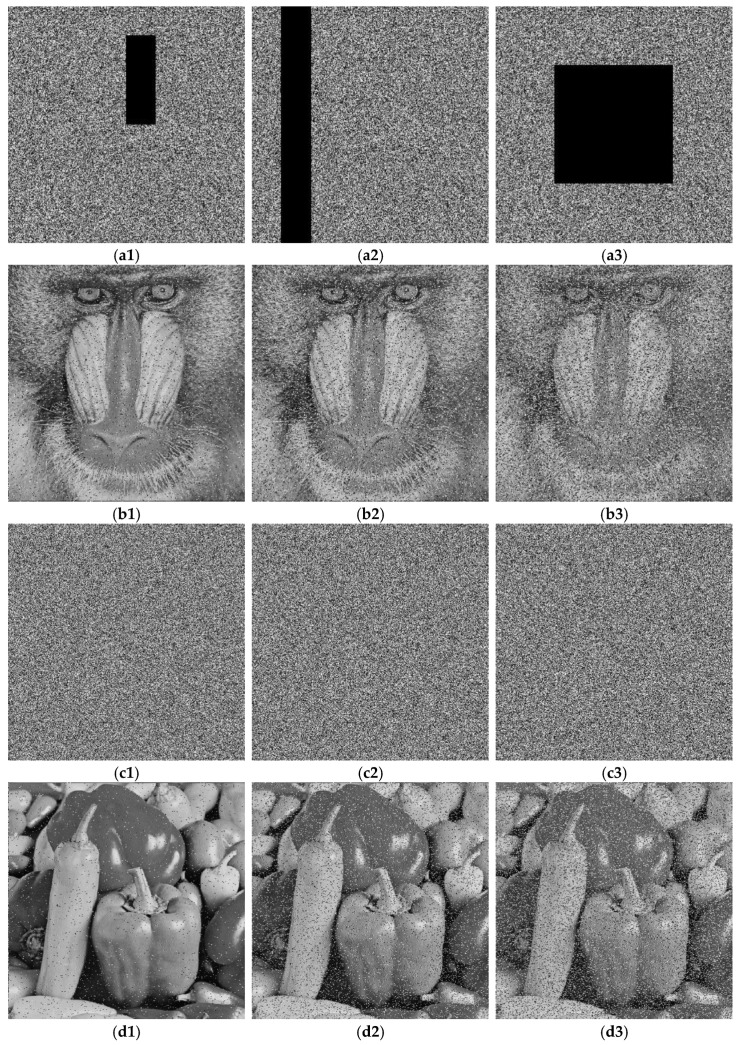
Analysis results of noise interference and data omission. Panels (**a1**–**a3**) illustrate the performance of the Baboon encrypted image under data omission scenarios of 1/64, 1/8, and 1/4; panels (**b1**–**b3**) depict the decrypted plaintext images corresponding to (**a1**–**a3**); panels (**c1**–**c3**) show encrypted picture data containing 1%, 5%, and 10% stochastic impulse noise; panels (**d1**–**d3**) present the decrypted plaintext images corresponding to (**c1**–**c3**).

During transmission through public channels, images could be affected by externally extra noise or data disappearance. To evaluate the resilience of the advanced algorithm in opposition to such disturbances, we introduce stochastic impulse noise introduced into the encrypted images or crop them before decryption. The outcomes are displayed in Figure 12(a1–a3). Panels (a1–a3) illustrate the performance of the Baboon encrypted image under data loss conditions of 1/64, 1/8, and 1/4, respectively; panels (b1–b3) show the corresponding decrypted plaintext images for (a1–a3), respectively; panels (c1–c3) depict the encrypted images with 1%, 5%, and 10% stochastic impulse noise, respectively; and panels (d1–d3) present the corresponding decrypted plaintext images for (c1–c3), respectively. These results indicate that the algorithm can successfully retrieve most of the essential information from images subjected to various levels of cropping. The results of the tests underscore the ability of the suggested encryption system to efficiently fend off noise attacks and endure the loss of data.

### 6.4. Computational Speed

In practical applications, due to hardware limitations, the computational efficiency and execution speed of image encryption algorithms are of great importance. Therefore, it is necessary to benchmark the computational efficiency of the image encryption process. To quantify these metrics, performance benchmarking was conducted on a workstation using MATLAB 2022b simulations in a controlled environment.

(1) Generate ten distinct encryption keys for encrypting the same test image (Baboon).
(2) Measure and record the encryption time required for each individual key.
(3) Compute the average encryption speed based on the ten recorded time measurements.
(4) Apply the identical procedure to determine the decryption speed through corresponding time measurements.

Furthermore, to evaluate computational efficiency, benchmarking was conducted using the encryption system constructed by THMHM under identical experimental conditions. Table 8 presents the computational rates (Mbit/s) of various similar encryption systems from recent years tested on a workstation configuration equipped with a 12th Gen Intel(R) Xeon(R) CPU E3-1230 v5 @ 3.40 GHz processor, 32 GB of RAM, and a Windows 11 64-bit operating system. Compared with other encryption systems, the encryption system of HTMHM achieves a higher computational rate.

### 6.5. Performance Comparison

To more clearly demonstrate the performance advantages of HTMHM, we selected recently constructed hyperchaotic maps from the latest literature and conducted a detailed comparative analysis of their chaotic characteristics. These characteristics include dynamic behavior, SE complexity, multistability, key dependency, frequency variability, histograms, autocorrelation analysis, and the NIST test suite. In Table 9, items that meet the evaluation criteria are marked with “√”, while those that do not are labeled with “-”. The comparative results show that the HTMHM not only has high computational efficiency but also exhibits higher spectral entropy complexity, making it more suitable for secure communication systems and encryption applications.

## 7. Conclusions

This study presents a novel 2D hyperbolic tangent memristive hyperchaotic map (HTMHM) with independent global and local amplitude control capabilities. The proposed system demonstrates rich behaviors, including bifurcation phenomena and coexistence attractors. Furthermore, it achieves superior nonlinear performance metrics, particularly when exhibiting SE complexity exceeding 0.9 under specific parameter configurations, outperforming other 2D maps. The numerical simulation results are further validated by experiments using an STM32 digital circuit setup, confirming the map’s practical feasibility. Moreover, the in-depth analysis of the HTMHM’s application in image encryption indicates that it possesses outstanding encryption performance, with high sensitivity to initial conditions and strong robustness against various attacks. Future work may focus on exploring additional applications of the HTMHM in other fields and further optimizing its performance for practical use.

## Figures and Tables

**Table 1:** The test results of the pseudorandom number sequences generated under the conditions *a* = 0.5, *h* = 1, *c*_1_ = 1, *k* = 2.654, IC = (0.1, 0.1), and IC = (0.1, 0.2) in the NIST suite.

Testing Item	*y*_0_ = 0.1		*y*_0_ = 0.2	
	*p*-Value*_T_*	Pass Rate	*p*-Value*_T_*	Pass Rate
Frequency	0.440975	251/256	0.534146	256/256
Block Frequency	0.377007	254/256	0.187581	255/256
Runs	0.440975	255/256	0.779188	255/256
Largest Runs	0.877083	254/256	0.344048	254/256
Rank	0.877083	251/256	0.171867	254/256
FFT	0.289667	253/256	0.397688	252/256
None-Ovla. Temp. ^1^	0.023545	253/256	0.018413	255/256
Ovla. Temp.	0.534146	250/256	0.691081	254/256
Universal	0.073872	253/256	0.988677	255/256
Linear Complexity	0.830808	253/256	0.948298	255/256
Serial (1st)	0.566688	255/256	0.801865	254/256
Serial (2nd)	0.131122	255/256	0.731886	253/256
Appr. Entropy	0.771469	256/256	0.053286	255/256
Cum. Sums (F)	0.017425	252/256	0.026233	255/256
Cum. Sums (B)	0.996335	251/256	0.344048	256/256
Ran. Exc. ^2^	0.108791	156/158	0.002337	149/149
Ran. Exc. Var. ^3^	0.001364	156/158	0.025807	146/149
Success Counts	15/15	15/15	15/15	15/15

^1^ The Non-Overlapping Template Matching Detection consists of 148 independent test units using the poorest-performing sub-item result as the final evaluation criterion. ^2^ The Random Walk Detection System is composed of 8 independent test modules, with the final determination based on the lowest evaluation indicator among all modules. ^3^ The Random Walk Frequency Detection Protocol performs a quantitative assessment of state transition frequencies through 18 test units, applying the global minimum value as the result determination principle.

**Table 2:** NPCR, UACI, and BACI results.

Test Item	Lena	Baboon	Peppers	Theoretical Value
NPCR	99.365%	99.3662%	99.2656%	99.6094%
UACI	33.2517%	33.2552%	33.4433%	33.4635%
BACI	26.4999%	26.6772%	26.7712%	22.7712%

**Table 3:** The results of the *χ*^2^ test.

Image	Lena	Baboon	Peppers	Theoretical Value
Plaintext	1.583 × 10^5^	1.836 × 10^5^	1.2017 × 10^5^	293.24783
Encrypted	249.7305	285.1191	283.4727	

**Table 4:** Comparison of information entropy for different encrypted images.

Image	Plaintext	Encrypted				
		HTMHM	Ref. [22]	Ref. [34]	Ref. [35]	Ref. [36]
Lena	7.4451	7.9993	7.9991	7.9991	7.9976	7.9991
Baboon	7.3583	7.9992	7.9990	7.9992	7.9973	7.9992
Peppers	7.5937	7.9992	7.9990	7.9989	7.9974	7.9990

**Table 5:** *MSE* and *PSNR* testing.

Image	MES	PSNR				
		HTMHM	Ref. [22]	Ref. [34]	Ref. [35]	Ref. [36]
Lena	34.3364	9.2272	9.2322	9.2421	9.2322	9.2322
Baboon	34.0647	9.5234	9.5466	9.8223	9.5466	9.4261
Peppers	34.6898	8.8933	8.9914	8.8535	8.9914	8.8962

**Table 6:** The correlation coefficients for each image across all directions.

Image	Direction	Plaintext	Encrypted				
			HTMHM	Ref. [22]	Ref. [34]	Ref. [35]	Ref. [36]
Lena	Horizontal	0.9809	0.0124	0.0231	−0.0084	0.0055	0.0037
	Vertical	0.9739	−0.0128	0.0138	0.0065	0.0305	−0.0004
	Diagonal	0.9625	−0.0153	−0.0002	0.0252	−0.0043	−0.0378
	Anti-diagonal	0.9689	−0.0358	0.0269	0.0121	0.0042	0.0058
Baboon	Horizontal	0.7661	0.0478	0.0060	0.0126	−0.0138	−0.0118
	Vertical	0.8625	−0.0100	−0.0324	−0.1311	−0.0267	0.0124
	Diagonal	0.7428	−0.0185	−0.0064	−0.0065	−0.0072	−0.0215
	Anti-diagonal	0.7064	0.0065	−0.002	−0.0364	−0.0187	−0.0314
Peppers	Horizontal	0.9809	−0.0066	0.0081	0.0314	0.0190	0.0099
	Vertical	0.9740	0.0039	−0.0126	−0.0035	−0.0029	−0.0049
	Diagonal	0.9652	−0.0188	−0.0097	0.0135	0.0334	0.0068
	Anti-diagonal	0.9595	−0.0487	−0.0064	−0.0026	−0.0217	−0.0245

**Table 7:** Key sensitivity and spatial analysis.

Var	Metric	Lena	Baboon	Peppers	Theoretical Value
*x*_0_	NPCR (%)	99.6099	99.6081	99.6097	99.6094
	UACI (%)	33.4656	33.4688	33.4640	33.4635
	BACI (%)	26.7772	26.7742	26.7690	26.7712
*y*_0_	NPCR (%)	99.1118	99.1103	99.1116	99.6094
	UACI (%)	33.2970	33.2964	33.2910	33.4635
	BACI (%)	26.6417	26.6365	26.6363	26.7712

**Table 8:** Computational speed comparison of encryption algorithms.

Encryption System	Encryption Speed (Mbit/s)	Decryption Speed (Mbit/s)
HTMHM	9.009	6.697
Ref. [22]	6.365	3.581
Ref. [34]	7.562	4.335
Ref. [35]	8.561	5.624
Ref. [36]	6.423	3.980

**Table 9:** Comparative analysis of performance characteristics.

Chaos Properties	HTMHM	Ref. [22]	Ref. [34]	Ref. [35]	Ref. [36]
***Characteristic analysis***					
Dimensionality	**2**	3	3	2	5
SE complexity (max)	**0.9523**	0.9246	0.9063	0.9134	0.9482
HCH	√	√	-	-	√
Multistability	√	√	-	-	√
Computational speed (Mbit/s)	**9.009**	6.365	7.562	8.561	6.423
***Security analysis***					
NIST statistical test suite	√	√	-	√	-
Key sensitivity	√	-	√	√	√
Histogram	√	-	√	√	√
Information entropy (Lena)	**7.9993**	-	-	7.9976	7.9993
MSE and PSNR	√	-	-	-	-
Autocorrelation analysis	√	-	√	√	√
Noise resistance	√	-	-	√	√

## Data Availability

The original contributions presented in this study are included in the article. Further inquiries can be directed to the corresponding authors.
